# Severe Hypothyroidism due to the Loss of Therapeutic Efficacy of l-Thyroxine in a Patient with Esophageal Complication Associated with Systemic Sclerosis

**DOI:** 10.3389/fendo.2017.00241

**Published:** 2017-09-20

**Authors:** Antonio Lobasso, Liliana Nappi, Letizia Barbieri, Carmela Peirce, Serena Ippolito, Debora Arpaia, Francesca Wanda Rossi, Amato de Paulis, Bernadette Biondi

**Affiliations:** ^1^Department of Translational Medical Sciences, Center for Basic and Clinical Immunology Research (CISI), University of Naples Federico II, Naples, Italy; ^2^Department of Clinical Medicine and Surgery, University of Naples Federico II, Naples, Italy

**Keywords:** hypothyrodism, sclerosis systemic, autoimmunity, hashomoto thyroiditis, l-thyroxine liquid formulation, systemic sclerosis

## Abstract

**Background:**

Thyroid function abnormalities and thyroid autoantibodies have been frequently described in patients with systemic autoimmune diseases as systemic sclerosis (SSc). Serum TSH levels are higher in SSc patients with more severe skin diseases and a worse modified Rodnan skin score. Asymptomatic esophageal involvement due to SSc has never been described as a cause of severe hypothyroidism due to l-thyroxine (l-T4) malabsorption in patients with Hashimoto’s thyroiditis (HT) and SSc.

**Case report:**

Here, we report a case of a 56-year-old female affected by both SSc and HT who developed severe hypothyroidism due to the loss of therapeutic efficacy of l-T4. Therapeutic failure resulted from the altered l-T4 absorption because of SSc esophageal complications. Clinical findings improved after the administration of oral liquid l-T4. Thyroid function completely normalized with a full clinical recovery, the disappearance of the pericardial effusion and the improvement of the pulmonary pressure.

**Conclusion:**

A recognition of a poor absorption is crucial in patients with hypothyroidism and SSc to reduce the risk of the subsequent adverse events. This case suggests the importance of clinical and laboratory surveillance in patients with SSc and HT because the systemic complications of these dysfunctions may worsen the prognosis of hypothyroid SSc/HT patients.

## Introduction

Systemic sclerosis (SSc) is a chronic, multi-system disorder of an unknown etiology characterized by abnormalities of the vascular system and complex alterations of the immune system, which eventually end up in fibrosis at variable degrees and specific organ damage. The severity and progression of the disease vary with heterogeneous clinical manifestations involving the main organs and districts such as the skin and the gastrointestinal tract (muscular atrophy and fibrosis) and a progressive and fatal visceral involvement of the kidneys, heart and lungs [pulmonary hypertension (PAH) and fibrosis] (1).

Hashimoto’s thyroiditis (HT) is an organ-specific autoimmune disorder associated with lymphocytic infiltration of the thyroid gland, leading to a progressive impairment of the thyroid function, and inducing subclinical and overt hypothyroidism (2). HT is frequently correlated with other organ and non-organ-specific autoimmune disorders (3). Many studies have evaluated the incidence of cases of thyroid autoimmunity and dysfunction in female patients with SSc, showing a high incidence of HT and new cases of hypothyroidism and thyroid dysfunction (4). The autoimmune regulator gene polymorphism has been linked to SSc/HT association (5), and autoimmune hypothyroidism seems to be associated with a higher index level of anti-Scl-70 (6). Serum TSH levels are higher in SSc patients with more severe skin diseases and a worse modified Rodnan skin score (7).

Interestingly, asymptomatic esophageal involvement due to SSc has never been described as a cause of severe hypothyroidism due to l-T4 malabsorption in patients with SSc/HT.

### Case Report

A 56-year-old woman was admitted to our department due to fatigue, cognitive-motor slowing and diffuse myalgias. She also reported weight gain (5 kg in the previous 2 weeks) and dyspnea.

In 2010, SSc was diagnosed and the patient suffered mainly from Raynaud’s phenomenon, sclerodactyly, digital tip ulcers, teleangiectasias, and interstitial lung disease. In 2012, a scleroderma renal crisis occurred. Finally, approximately 20 years before our observation, HT had been diagnosed and treated with l-thyroxine (l-T4) replacement therapy with a good and stable control of hypothyroidism with a dose of 125 µg (2 µg/kg/day). Thyroid function tests were normal the last 3 months but suddenly worsened (Table 1). The patient was hospitalized for the rapid onset of severe hypothyroidism despite the treatment with l-T4.

**Table 1:** Thyroid laboratory values before, during, and after the hospitalization.

Timing	TSH (n.v. 0, 3–4, 2 mIU/l)	FT3(n.v. 2, 0–4, 4 pg/ml)	FT4(n.v. 0, 9–1, 7 ng/dl)
l-thyroxine (l-T4) tablet therapy (125 μg/day), before the hospitalization	1.7	3.1	1.3
l-T4 tablet therapy (125 μg/day), during the hospitalization	387	0.5	0.3
l-T4 tablet therapy (150 μg/day), during the hospitalization	365.1	1.4	0.7
l-T4 oral drops therapy (150 μg/day), during the hospitalization	115	2.1	1
l-T4 oral drops therapy (150 μg/day), 3 months after the hospitalization	2.1	3.3	1.1

During the hospitalization, thyroid function tests and ultrasonography were repeated. Doppler echocardiography, chest tomography, and a complete diagnostic evaluation for malabsorption [including esophagogastroduodenoscopy (EGD) and esophageal manometry] were also performed.

### Results

**Figure 1:**
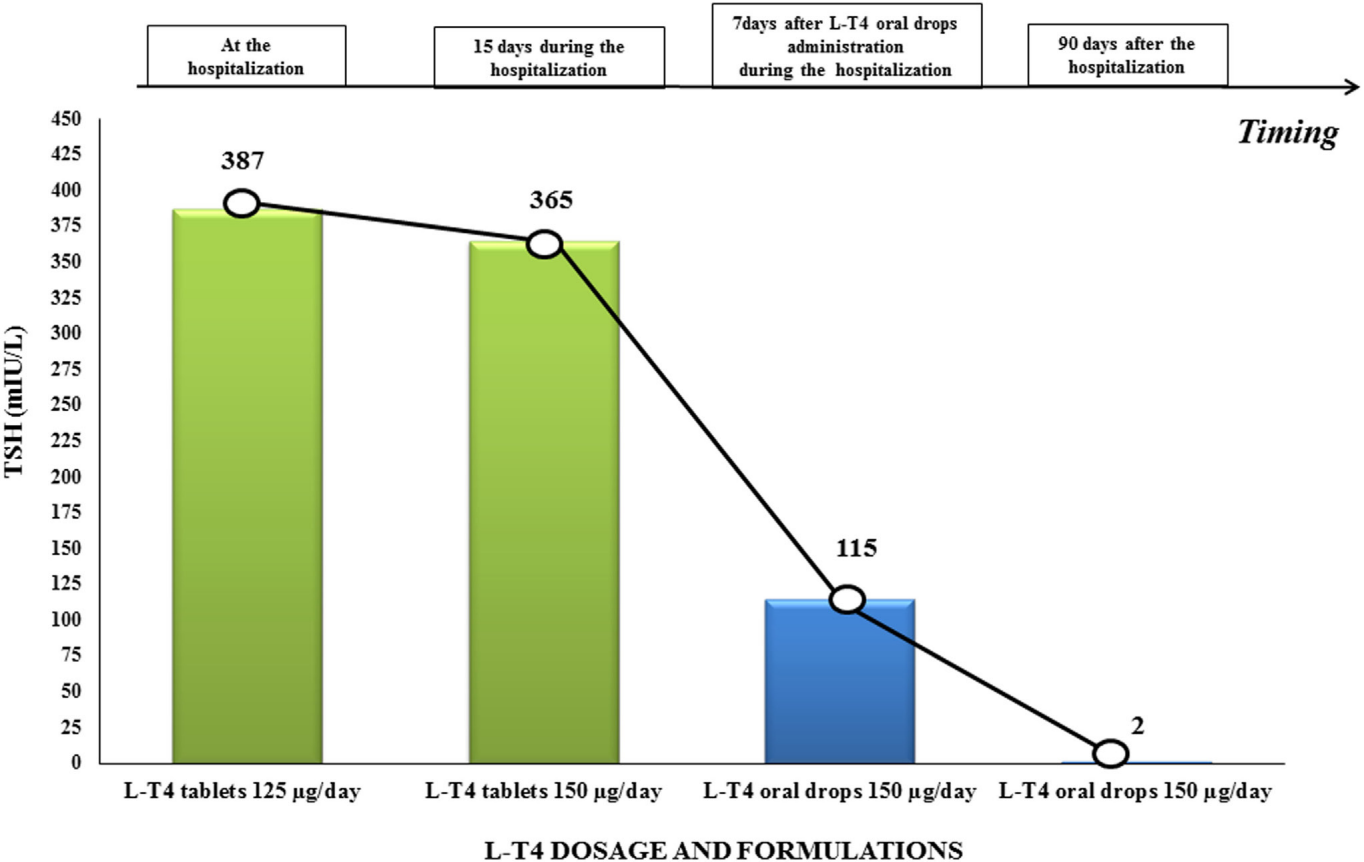
TSH values before and after switching l-thyroxine (l-T4) therapy from tablets to oral drops in our patient with systemic sclerosis-related malabsorption. TSH levels increased up to 387 μU/ml. during l-T4 tablets. Oral drops were administered from day 4 and TSH values progressively normalized after 3 months.

Sinus bradycardia (50 beats/min) was noticed on physical examination and was confirmed by electrocardiogram. Hypothermia (body temperature was about 35°C) and cool pale skin were also detected. The laboratory results confirmed severe hypothyroidism: TSH 387 mIU/l (0.3–4.2 mIU/l), FT3 0.5 pg/ml (2.0–4.4 pg/ml), FT4 0.30 ng/dl (0.9–1.7 ng/dl), CPK was 654 U/l (29–168 U/l), and antithyroid peroxidase (364 IU/ml) and anti-thyroglobulin antibodies (>4,000 IU/ml) were elevated (Table 1; Figure 1). Thyroid ultrasonography showed a typical HT inhomogeneous glandular pattern with absent Power Doppler signal. Serum ACTH, cortisol levels, and the 24-h urinary excretion of free cortisol were within the normal range. Hemoglobin, sodium, potassium, calcium and phosphorus levels, iron status, blood glucose, albumin, vitamin D, total protein, amylase, and electrophoretic protein pattern reported normal values. Doppler echocardiography displayed a pericardial effusion (about 15–35 ml) and a reduction of the ejection fraction with a worsening of the pulmonary hypertension (PAP 30 mmHg). Myxedema coma was excluded despite the severity of the symptoms for the lack of neurological involvement.

We increased the l-T4 dosage to 150 μg/day. Medications interfering with l-T4 metabolism or absorption were not added during the hospitalization and the patient continued a regular diet. However, thyroid laboratory results did not improve during the following weeks (Table 1; Figure 1). We excluded the poor patient’s compliance, and a correct adherence to l-T4 therapy was assessed during the hospitalization. Atrophic gastritis, celiac disease, and other causes of malabsorption were excluded. EGD (performed after 12 h of fasting overnight), showed a typical pattern of mild chronic gastritis not associated with *Helicobacter pylori* infection. Esophageal manometry showed a severe distal esophageal dysmotility with a slow progression of the bolus to the stomach.

**Figure 2:**
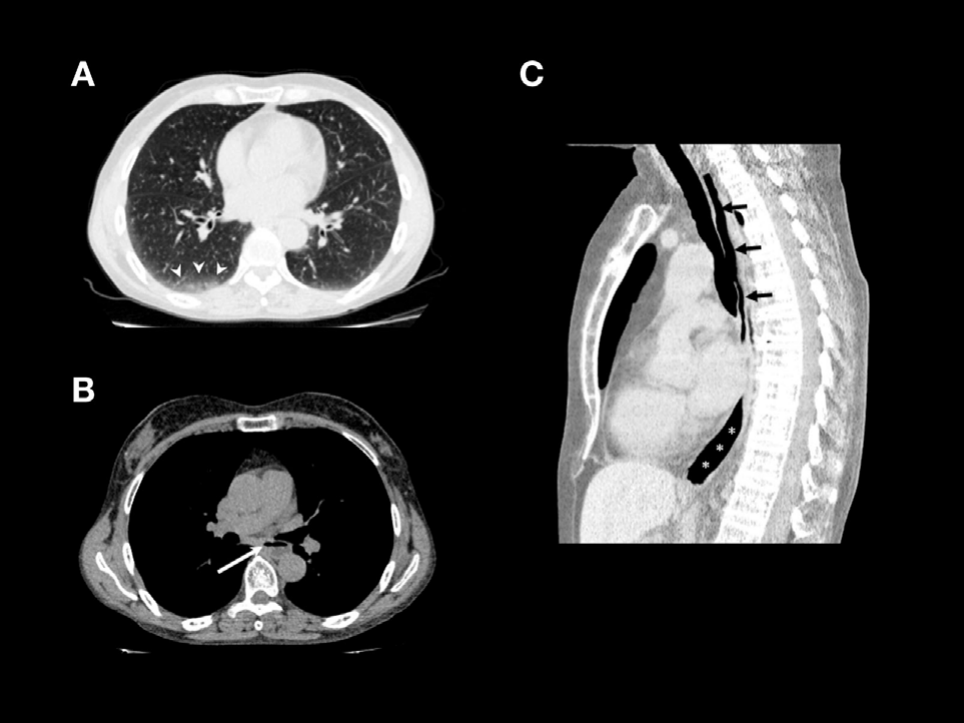
**(A)** Non-contrast chest CT (lung window) shows interlobular septal thickening and ground-glass opacities in the immediate subpleural lung with peripheral and lower lobe predominance, especially on the right side (arrowheads). **(B)** Non-contrast chest CT (mediastinal window): air–fluid level due to stasis in thoracic esophagus (arrow). **(C)** Non-constrast chest CT, sagittal reformation: air-filled hypotonic upper (arrows) and lower (asterisks) thoracic esophagus.

Interestingly, a chest tomography (Figure 2A), taken to exclude a respiratory exacerbation, revealed the presence of liquids and food stuck in the esophageal lumen (Figures 2B,C).

We switched the same dose of l-T4 tablets to liquid drops and administered 42 drops daily of l-T4, which is the equivalent of 150 µg of l-T4 tablets. A few days after initiating the solution therapy, clinical findings improved in terms of fatigue and drowsiness. One week later, the laboratory tests improved (Figure 1; Table 1); heart rate was 62 beats/min and body temperature improved (36°C). During the subsequent follow-up, thyroid function completely normalized (Table 1) and we observed a full clinical recovery, the disappearance of the pericardial effusion and an improvement of the pulmonary pressure.

## Discussion

Thyroid hormone deficiency, when severe and untreated, may be a life-threatening condition especially in patients with comorbidities such as SSc. Although all of the organs and main systems can be interested in this disease, PAH is the major cause of mortality in patients with SSc (8). The onset of autoimmune hypothyroidism can worsen the prognosis of PAH in SSc (9, 10).

The gastrointestinal tract is most frequently involved in SSc. The diagnosis of gastrointestinal sclerosis may be delayed because of the absence of clinical symptoms despite the organ damage. However, the consequences of the drug-impaired absorption and their reduced bioavailability are often dangerous, especially in patients with comorbidities receiving oral drugs (11).

l-Thyroxine, a lifelong treatment in patients with persistent hypothyroidism, is generally orally administered; an empty stomach and a correct storage of l-T4 is necessary (12, 13). A concomitant administration of other medicaments and supplements should be avoided to permit a good absorption of l-T4 (11–14).

In our patient with SSc/HT, we observed the onset of severe and acute hypothyroidism due to l-T4 tablet malabsorption for the esophageal sclerosis. We can hypothesize that the l-T4 tablets were not well absorbed in our patient because: (1) they did not completely reach the stomach and/or had an inadequate dissolution phase (since they did not find the acid pH necessary for their optimal dissolution) or (2) were sequestered by the food abnormally stuck in the esophagus.

More important, our patient reported very mild gastrointestinal symptoms without dysphagia despite the severe alteration of the esophagus motility detected by manometry examination. In fact, SSc determines only an alteration of esophageal motility without the impairment of esophageal mucosa; however, this alteration was able to impair the bolus progression and reduce the l-T4 absorption in our patient. We excluded all of the other causes of l-T4 malabsorption, poor-compliance and incorrect l-T4 administration and concluded that the reduced efficacy of the l-T4 tablets was secondary to SSc esophageal complications.

Current guidelines recommend oral l-T4 in tablets as treatment of choice in patients with hypothyroidism (11). However, liquid formulation of l-T4 is available in Italy (Tirosint drops, distributed by Ibsa Farmaceutici Italia Srl); it is solubilized in ethanol without other excipients, and therefore, better absorbed compared to the tablet form, allowing a quicker absorption of l-T4 without a dissolution phase (15, 16). The literature data have reported positive effects of l-T4 liquid solutions compared to l-T4 tablets in presence of drug interference, food, beverages, gastrointestinal disorders, and/or other factors interfering with l-T4 absorption (11–19). Our patient was unresponsive to oral l-T4 tables despite the increase in the l-T4 dosage. On the contrary, a normalization of thyroid function was achieved when l-T4 formulation was switched to the oral drops in an equivalent dosage.

## Concluding Remarks

It is essential to assess thyroid function as part of the clinical screening in the SSc patients due to the high prevalence of HT and antithyroid autoantibodies in presence of systemic autoimmune disease. This case suggests the importance of clinical and laboratory surveillance in patients with SSc and HT because SSc esophageal complications can decrease the efficacy of l-T4 therapy and persistent hypothyroidism can worsen the prognosis of these patients. A periodic follow-up is mandatory to assess the adequacy of l-T4 replacement therapy, considering the possible onset of asymptomatic esophageal complications of SSc.

## Ethics Statement

The patient gave written informed consent for the publication of this case report.

## Author Contributions

All the authors contributed in writing the manuscript, revised and approved the final version, and agreed to be accountable for the content of the work.

## Conflict of Interest Statement

The authors declare that the research was conducted in the absence of any commercial or financial relationships that could be construed as a potential conflict of interest. The handling editor shares an affiliation with the reviewer, SF.

## Abbreviations

EGD, esophagogastroduodenoscopy; HT, Hashimoto’s thyroiditis; l-T4, l-thyroxine; mRSS, modified Rodnan skin score; PAH, pulmonary hypertension; SSc, systemic sclerosis.
